# A Multiple Streams Approach to Understanding the Issues and Challenges of Lyme Disease Management in Canada’s Maritime Provinces

**DOI:** 10.3390/ijerph16091531

**Published:** 2019-04-30

**Authors:** Mario Levesque, Matthew Klohn

**Affiliations:** Department of Politics and International Relations, Mount Allison University, 144 Main St., Sackville, NB E4L 1A7, Canada; mdklohn@mta.ca

**Keywords:** Lyme disease, ticks, health policy, Canada, New Brunswick, Nova Scotia, Prince Edward Island, politics, policy change, multiple streams

## Abstract

This study examines potential challenges facing Lyme disease patients in Canada’s Maritime provinces—New Brunswick, Nova Scotia, Prince Edward Island—and considers how issues could be addressed. Reviews of both the academic and grey literature are complemented by surveys targeting both medical professionals and decision makers in government. Combined, the literature reviews and surveys demonstrate that there is considerable debate surrounding the effectiveness of testing, treatment options, and the existence of chronic Lyme disease. As the focus on the Maritimes demonstrates, these debates often pit the medical community against patients and patient advocates and, thus far, governments have been unable to produce policy that entirely pleases either side. Moving forward, this study recommends the creation of a discussion forum via a federal Commission of inquiry to review best practise guidelines for Lyme disease. The key is to foster an unbiased probe of central issues surrounding treatment and diagnosis without alienating stakeholders. This course of action will not necessarily solve the issue of Lyme disease, but would foster a greater understanding through dialogue that includes and validates the experiences of stakeholders, which is something that is currently missing.

## 1. Introduction

Lyme disease has increased in prevalence in Canada in recent years. As a vector borne disease, Lyme disease is transmitted to humans via the bite of *Borrelia burgdorferi* infected *Ixodes scapularis*, commonly known as black-legged ticks. These ticks have expanded into Canada, in particular Canada’s Maritime provinces of New Brunswick (NB), Nova Scotia (NS) and Prince Edward Island (PEI), largely due to climate change. The result has been a dramatic rise in Lyme disease [1]. Early diagnosis and treatment are essential, yet physicians’ reluctance to do so has contributed to the struggle within Canada’s health care system to adequately address this disease leading many Canadians to seek treatment in the United States (U.S.), which is costly and complicated [2]. For example, prescriptions are seized at the border upon returning to Canada and cannot be filled in Canada [3]. It is this tension between patients, physicians and the health care system that is at the heart of our investigation as it seeks to answer the following questions: What are the systemic blockages facing patients surrounding Lyme disease diagnosis and treatment within Canada’s Maritime provinces, and how can government address these issues?

We approach the questions in three ways. First, a review of the literature, both academic and grey, is conducted to illustrate the current scholarship surrounding Lyme disease in order to better understand why Lyme disease has become such a polarizing issue. The key debates revealed include the current testing procedures for diagnosis and the length and efficacy of antibiotic prescriptions. Combined, these issues contribute to a contentious discussion, which often pits the majority of the medical community against patients and Lyme disease advocacy groups. Moving forward under such conditions is challenging. Second, surveys of family physicians, politicians and government health officials within the Maritime provinces were conducted to better understand how Lyme disease is processed and perceived. This allowed us to situate the regional context in the broader literature to reveal potential blockages. Third, we propose policy options in the analysis section that follows. These options were informed through the lens of multiple streams theory, which looks to explain the necessary circumstances for policy change to be achieved via the coming together of problems, solutions and politics by policy entrepreneurs within a favourable window of opportunity. We conclude that an unbiased yet guided discussion forum to investigate best practise options, a Commission of inquiry that includes all stakeholders, may be a good means of bridging the gap between advocacy groups, governments and the medical community before innovations in the realm of diagnosis and treatment options can be realized. 

### 1.1. Current Scholarship Surrounding Lyme Disease

#### 1.1.1. The Academic Scholarship

Increased scholarship over the past twenty years has been dedicated to the study of Lyme disease in North America. This scholarship has centred on the expansion of ticks in Canada, diagnosis issues, treatment options and chronic Lyme disease. 

##### Expansion of Ticks in Canada

At the heart of understanding tick expansion is recognizing the nature of the disease itself. The main cause (though not the only one) of Lyme disease in North America is the bacterium *Borrelia burgdorferi*. This bacterium is transmitted to humans via the bite of infected ticks, most commonly *Ixodes scapularis*, better known as the black-legged or deer tick [4]. These ticks spread by attaching themselves to animals or humans. Migratory birds, in particular, allow ticks to move great distances and allow the disease to spread across wide geographic areas [5]. Lyme disease is widespread in the United States with over 20,000 cases reported each year in 2009 growing to over 30,000 cases per year in 2018 [1,6]. However, as the range of *I. scapularis* expands, more areas are at risk for Lyme disease.

Agreement in the literature exists on the fact that *I. scapularis* has been expanding due to climate change, as warmer weather has allowed their range to gradually expand north. Previously established tick populations thrived in two regions of the U.S.: The Northeast, particularly in the states of Maine, New Hampshire, New York, and Massachusetts; and, the Midwest, particularly in Minnesota and Wisconsin [7]. As the range for ticks expands northward, Canada is projected to have more cases of Lyme disease [1]. This is especially true for Canada’s Maritime provinces given their proximity to the Northeastern U.S. This increase can already be seen, as reported cases of Lyme disease have risen from 144 in 2009 to 917 in 2015, with the largest increase in a geographic area being found in Nova Scotia [8]. Model projections further predict that the situation will worsen as climate change continues. One study, for example, projects that tick populated areas will expand 46 km per year for the next decade within Eastern Canada (defined as east of Manitoba) and that the proportion of people living in these areas will rise from 18% in 2010 to 80% by 2020 [5]. 

##### Diagnosis

A similar convergence, however, does not exist surrounding the diagnosis of Lyme disease. There are two means by which an individual can be diagnosed with Lyme disease, either clinically or via serological testing. A clinical diagnosis can take place if an individual shows erythema migrans and has plausible exposure. That is, exposure to known areas with high tick populations instead of the clinical condition of the patient [1]. Some, such as Andany et al. [9], are adamant on the need for the presence of erythema migrans for a positive diagnosis; however, over-relying on its presence is problematic due to its wide variation in those with Lyme disease. The result is that Lyme disease may be underreported.

If the patient does not have erythema migrans then they must receive positive serological tests for evidence of antibodies against the Lyme disease bacteria (in most cases even if they have been exposed to areas of high tick populations). Canada follows the Centers for Disease Control and Prevention (CDC) protocol for serological testing by first, the Enzyme Immunoassay (ELISA) test, and, if positive, verification via a Western blot test. These tests look for antibodies the body produces in response to infection [9]. As a result, they have low sensitivity in detecting early Lyme disease because the immune response is in its early stages of development at this time. Therefore, serological testing for early Lyme is notorious for a high rate of false negatives due to its estimated sensitivity of only 40% [9]. This compares to much higher sensitivities (reported as high as 87% depending on which commercial test is used) if the tests are done between weeks 4–6 when antibodies are normally produced (disseminated infection) and sensitivity to the two-step protocol increases significantly [9]. Due to challenges with diagnosing early Lyme disease, physicians are advised not to proceed to serological testing for patients with nonspecific symptoms [9]. 

Current and former patients are unhappy with diagnosis procedures with many seeking treatment outside of the conventional Canadian health care system. Issues abound including perceived misdiagnosis, failure to obtain a diagnosis, or persistence of symptoms often after years of seeing specialists [10]. Some scholars have also been critical of the current two-tier testing system for Lyme disease in Canada [10,11,12]. For example, Ogden et al. have hypothesized that the location and or the time of year when an infection is contracted may display variances in Lyme disease itself. They concluded that Lyme disease in Canada may vary between Eastern and Western Canada, dividing the country on the Ontario–Manitoba border, possibly due to the disease descending from two different strains [11]. Lloyd and Hawkins also hypothesize that these geographic variations may account for some of the under-reporting of the disease, as some variations may go undetected with the current two-tier serological testing [13]. While improvements to diagnosis procedures have been suggested, changes have yet to materialize [11,12].

##### General Physician Knowledge

Complicating matters is the minimal knowledge of Lyme disease by medical professionals. For example, in examining the experiences of individuals who sought treatment for Lyme disease outside of the conventional Canadian health care system, Boudreau et al. reported many individuals struggling to get treatment in Canada or be successfully diagnosed with Lyme disease while others were denied conventional antibiotics and belittled for insisting that they had Lyme disease [10]. This frustrates Canadian Lyme disease advocacy groups who argue that there is not enough guidance about how to treat individuals who are bitten by ticks [14]. However, once these individuals moved outside of the conventional health care system, their experience changed, with physicians generally being more receptive to the possibility of Lyme disease and less likely to stigmatize patients [10].

Seeking medical treatment outside the Canadian health care system, and specifically in the United States, remains controversial. Much of the controversy surrounds the current two-tier serological testing procedure where it is argued that it is no more effective in the U.S. than that found in Canada given similarly high false negative rates in the U.S. (e.g., 57%) [4]. Canadian physicians continue to follow the CDC protocol given its track record and point to the fact that U.S. laboratories are not required to do so, thus potentially increasing the chances of false positives or negatives. For example, the CDC only uses the Western blot test as a second test for verification while some U.S. specialty laboratories for detecting infection with *B. burgdoferi* only rely on this test, which on its own has a high false positive rate [4]. Furthermore, a Western blot test is not a simple yes or no test. It needs to be interpreted, for which the CDC sets strict criteria; however, U.S. labs use in house criteria that may not be as reliable as the CDC protocol used in Canada [9]. A complicating factor is the fact that many physicians are also hesitant to diagnose Lyme disease without specific symptoms such as erythema migrans given that other causes are more common [9,15]. The point is that physicians and patients would both like a simple and invariant test which, at this time, is not supported by the biology [16].

Canadian physicians generally agree that seeking Lyme disease testing in the United States is a mistake as misdiagnosis and or treatment could lead to lasting economic, psychological, and physically adverse outcomes [4]. Ironically, many individuals experience these adverse outcomes as a result of the current means by which Lyme disease is addressed in Canada. In some cases, individuals feel all together abandoned by the Canadian health care system. In the words of one patient, “The last straw was when my last neurologist told me there was nothing more that could be done for me and that I would have this unexplained pain for the rest of my life [...] I felt like I was dying [...] I was feeling weak emotionally and physically, and I felt that the specialist had closed the doors on me” [10]. Patients are thus left in a precarious position—dismissed and alienated by their own health care system while chastised for seeking treatment elsewhere [10]. By refusing to entertain the possibility of a Lyme disease diagnosis, the majority of the medical community is forcing adverse outcomes on the very people they are trying to help.

##### Treatment

Debate is not limited to the diagnosis of Lyme disease, but extends to treatment options. Lyme disease is currently treated through a variety of short-term (10–28 days) antibiotics which are widely accepted within the academic and medical communities [17,18]. However, some patients and medical professionals argue for longer antibiotic treatment periods due to the return of symptoms after the use of short-term antibiotics, which has been termed chronic Lyme disease [19,20,21,22]. The majority of the medical community contests the existence of chronic Lyme disease for a variety of reasons. Contestation surrounds classification issues (post vs. late Lyme disease), symptom manifestation, associated test results (positive/negative) and treatments [19,23]. Many physicians are also generally skeptical of individuals who believe they have chronic Lyme disease and seek treatment for it because many are found to be suffering from different illnesses [19]. However, given the limitations of current testing procedures, Lyme disease is not necessarily ruled out. Moreover, simply renaming the condition as chronic fatigue syndrome (CFS) or fibromyalgia due to the biological limits of test sensitivity is not helpful [16]. Lastly, physicians are also widely opposed to the long-term use of antibiotics based on perceived medical risks [24].

Challenges surrounding diagnosis and the debate over the existence chronic Lyme disease, and by extension the use of long-term antibiotics, has driven many Canadian patients into seeking treatment outside of the conventional health care system. Surprisingly, Boudreau, Lloyd, and Gould found that patients were “pushed” to seek out-of-country treatment given specialists in Canada, particularly infectious disease experts and neurologists, were the most unhelpful within the Canadian health care system. Changes are required including an effective treatment protocol, effective diagnostic tests, more knowledgeable physicians, financial coverage for alternative treatments, as well as increased respect towards patients, including acknowledgment of the disease itself and the suffering state of the patient [10]. This study demonstrates just how disenfranchised many people feel within their own domestic health care system. More has to be done to bridge this gap concerning treatment if progress is to be made.

Current challenges and debates have also renewed interest in Lyme disease vaccines. Two vaccines, approved in the 1990s, were voluntarily withdrawn from the market under the threat of a class action lawsuit due to adverse events to the vaccines (although U.S. Federal Drug Administration inquiries found the adverse events to be within the range of other vaccines) [25]. Overcoming public resistance going forward will be especially difficult within the current strong anti-vaccine movement climate [26,27,28,29].

Overall, the divergence within the academic community regarding Lyme disease is shrinking with increasing research. The facts are that, first, Lyme disease is an infectious disease that infects humans via *B. burgdorferi* and is transmitted to humans via ticks (most often black-legged or deer ticks). Second, increasing temperatures due to climate change are facilitating the expansion of tick populations into Canada, giving rise to more cases of Lyme disease. How to address this disease remains a topic of heated debate. While the medical community generally agrees on the diagnosis process and short-term antibiotic treatment, much criticism exists from some scholars and, particularly, patients and patient advocates. The main issue at the root of these debates is the accuracy of serological testing to determine Lyme disease [11,12]. Many Canadians who continue to experience symptoms often doubt their test results due to their high inaccuracy, especially in cases of early Lyme disease. At the same time, Canadian physicians are extremely wary of serological testing being conducted in the U.S. due to the lack of set protocols. Finally, the inaccuracy of serological testing fuels the debate over chronic Lyme disease, as most physicians claim the infection does not persist after short-term antibiotics while patients and patient advocates counter that serological tests are simply inadequate at detecting the infection. The challenge is in how to move forward, especially in relation to public policy [30,31]. 

#### 1.1.2. The Grey Scholarship

##### Media Sources

Media coverage surrounding Lyme disease in Canada generally covers similar topics—the expansion of tick populations and by extension Lyme disease, government initiatives, as well as cases of mistreatment. The media is also largely sympathetic towards Lyme advocacy groups and those suffering from Lyme disease, a position that is not held in the majority of the academic scholarship [10].

Canadian news articles typically report an increase in tick populations and by extension cases of Lyme disease. Within the Maritimes specifically, the rise of Lyme disease in Nova Scotia draws much attention due to the rapid increase of infections—from 7 confirmed cases in 2007 to 326 in 2016 [32]. Rankin notes that in 2016 Nova Scotia’s rate of Lyme disease was 12.7 times higher than the national average with 34.4 cases per 100,000 people [33]. It is also in stark contrast to the neighbouring provinces of New Brunswick and Prince Edward Island with 1.5 and 2.7 cases per 100,000 people respectively [34]. However, the spread of ticks into these provinces will likely lead to an increase of reported cases of Lyme disease [35,36,37]. This rapid rise of Lyme disease is characteristic of the news coverage and consistent with that found in the academic scholarship in that the rise is connected to climate change [38,39].

Some of the most common media stories concerning Lyme disease in New Brunswick, Nova Scotia and PEI surround individuals who have struggled to find treatment for symptoms they attribute to Lyme disease [40]. The common theme is that the patient is struggling to find a doctor who will address their condition, demonstrating a lack of confidence in the health care system’s current approach to the condition. For example, Amanda Millar, a resident of PEI, was shown to be negative on an ELISA test, leading health professionals to rule out Lyme disease. However, she has since paid $4000 out of pocket to travel to British Columbia to see a doctor who would consider treating her for Lyme disease [40]. Such an expensive trip is not uncommon as the *Telegraph Journal* covered the case of Mitch Thibodeau whose doctor had retired, forcing him to travel the country in search of another physician who would consider the possibility of antibiotic treatment until symptoms resolved [41]. An article from the *Chronicle Herald* echoed these two stories, with the patient, Jana Young from Nova Scotia, being forced to search for a doctor willing to consider treating her for Lyme disease [33]. All of these articles concerning patient struggles portray the patient in a sympathetic manner, leaving the reader with questions about the current state of Lyme disease treatment in Canada. 

Media reports also illustrate the significant divide that exists between the majority of the medical community, patients and patient advocates. Ubelacker illustrates this debate concerning the number of Lyme disease cases in Canada quoting Harvey Artsob, the Director of zoonotic diseases at the Public Health Agency of Canada, that, annually, there are between 20 and 60 cases of disseminated Lyme disease every year. The Canadian Lyme Disease Foundation has argued that, in reality, the infection rate was between 2000 and 20,000 cases, demonstrating the vast gap between the two positions [41]. 

Anne Kingston also focuses on this tension, in particular how patients increasingly attempt to have a say in their own treatment [42]. She notes how Lyme disease is indebted to patient advocacy as the disease was originally discovered largely due to the efforts of Polly Murray and Judy Mensch, two mothers who gathered a database of cases, which allowed researchers to discover Lyme disease. Kingston also documents patients’ struggles with the health care system, with specific references to physicians claiming an individual’s symptoms as psychosomatic. The question of whether patients should have an equal role in influencing health care policy as the medical community and researchers is explored and remains controversial. For example, patients and advocacy groups were angered that they were not consulted for Bill C-442, *An Act respecting a Federal Framework on Lyme Disease*, introduced in 2011 and entered into law in December 2014, illustrating the potential shortcomings of patient advocacy [42]. Overall, Kingston seems to establish sympathy for these patients supporting that more needs to be done but seems skeptical about patients having an equal voice in informing health care policy. 

##### Government Documents

Increased media and public attention and academic scholarship has resulted in some government action. The Canadian federal government and the Maritime provinces, except PEI, have released similar policy statements and developed broad frameworks to address Lyme disease. Provincial efforts are at times limited and refer individuals back to federal initiatives such as in New Brunswick’s case. These frameworks typically note that Lyme disease is on the rise and projected to continue expanding and, in the federal government’s case, that Lyme disease became a nationally notifiable disease in 2009 [30,43]. To support these claims, governments cite the increase in confirmed cases of Lyme disease illustrating how the disease is distributed across the country. Nova Scotia, for example, has a high rate of Lyme disease especially when compared to the other Maritime provinces. In 2016 alone, there were 326 confirmed cases of Lyme disease in Nova Scotia, an increase from 247 cases in 2015 [44]. This is considerably higher than New Brunswick which only recorded 11 cases in 2015 and 8 cases in 2016 [43]. Data for PEI is lacking however. This could partially be due to the idea that PEI is untouched by Lyme disease due to the province’s lack of deer, which some assume means no deer ticks or Lyme disease. However, ongoing research has demonstrated that this is a misconception as through infected ticks and dogs, and by extension humans, Lyme disease is becoming more prevalent in PEI [37]. Furthermore, the above data should be seen as conservative given the fact that Lyme disease is often misdiagnosed and underreported [13]. 

Canada’s *Federal Framework on Lyme Disease* consists of surveillance, education and awareness, as well as guidelines and best practises [30]. The governments of Nova Scotia and New Brunswick follow a similar approach. In the Nova Scotia *Tick Borne Response Plan 2018*, the Nova Scotia Department of Health and Wellness is charged with surveying and reporting on areas with tick populations [45]. It is also tasked with developing guidance documents for public health surveillance and tick-borne case management, reflecting best practise and surveillance aspects in their approach. Public education efforts are also prioritized [45]. The *New Brunswick Lyme Disease Strategy 2017* has the same core aspects concerning surveillance and public education, while referencing a medical practitioner survey in creating an informational document to reinforce best practises [43]. The outlier among these governments is PEI, which has yet to release a framework or plan. The only PEI government publication that could be found was an algorithm for diagnosing the disease, which was simply a flow chart that laid out signs and symptoms of Lyme disease [46]. PEI is considerably behind its Maritime neighbours in terms of Lyme disease management. 

Government efforts have not been well received by patient advocacy groups. Tension surrounds the current testing protocol and guidelines regarding Lyme disease diagnosis and treatment (use of short-term antibiotics). Government action plans seem to reinforce practises that advocacy groups contest. This is exemplified in the *Statement for Managing Lyme Disease in Nova Scotia* which illustrates how NS favours conventional approaches supporting the current CDC-endorsed two-tier testing method and the current treatment of antibiotics, stating that 95% of Lyme disease cases are cured via short-term antibiotics [47]. It also urges individuals not to seek other forms of testing except the current two-tiered system, which is another slight to Lyme disease patients and patient advocates, many of whom feel they were forced to seek alternative testing and treatment due to adverse experiences within the conventional health care system. The Nova Scotia approach is also not necessarily surprising given the actions of its Chief Medical Officer of Health, Dr. Robert Strang, whose recent tweet dismissed chronic Lyme disease as a pseudoscience supported by a cult following [48]. Such an action by the province’s top health official is dismissive, demonstrates a lack of understanding, and questions the efficacy of the province’s Lyme disease strategy [49]. Strang’s statement only fueled patients’ and patient advocates’ anger, similar to what occurred when they were not consulted when the federal framework was crafted. This ultimately questions the role patients should have in health care policy formulation [42].

Overall, the grey literature illustrates some of the shortcomings of the current approach to Lyme disease. Governments have accepted the wide range of scholarship, which indicates a dramatic rise in ticks and Lyme disease that is projected to continue. Frameworks and action plans have been created and, in the case of the federal government, funds have been allocated for Lyme disease research. However, governments have also strongly sided with the majority of the Canadian medical community, reinforcing the current approach to Lyme disease. Even so, some do recognize the tension between patients and the medical community. For example, the *New Brunswick Lyme Disease Strategy* acknowledges that Lyme disease has become a political issue with advocacy groups working to have patient experiences heard and advocating for better diagnosis and treatment [44]. Yet, government efforts have done little to diffuse this tension, as their frameworks and plans do not address the current diagnostic procedures and treatment options that frustrate Lyme disease patients and patient advocates. Presently, governments seem only willing to recognize their pleas for better Lyme education among physicians. Yet, if educating physicians primarily consists of reinforcing the status quo, patients and patient advocates will see these efforts as insufficient and tensions will continue. 

## 2. Materials and Methods 

Our study focused on Canada’s Maritime Provinces—New Brunswick, Nova Scotia, Prince Edward Island—and relied on two surveys (see Supplementary Materials) designed to answer the central research questions of this project: What are the systemic blockages facing patients of Lyme disease within the Maritime Provinces and how can government address these issues? The project and surveys were reviewed and approved on 25 May 2018 (Research Ethics Protocol #102206) by Mount Allison University’s Research Ethics Board prior to their administration.

### 2.1. Terminology

For the purposes of this research, the conventional (or traditional) Canadian health care system refers to Canada’s publicly funded health care system. Treatment outside of this system, such as unauthorized medical visits to the United States, is considered as an alternative to Canada’s conventional health care system.

### 2.2. Survey Development and Administration

Two electronic surveys were designed for distribution using Survey Monkey, a web-based survey company located in the USA. Survey Monkey was used due to its minimal cost and ease of use. The surveys combined questions offering a range of answers for participants to answer using a Likert scale, questions that asked them to rank a series of choices, and questions to select a particular choice (e.g., province of residency). The first survey focused on family physicians and nurse practitioners given they are the first point of contact for patients. Questions probed their perceptions of Lyme disease, general knowledge, professional approach to the disease, and how to improve services. 

The second survey was sent to provincial politicians and government health officials such as public health officers, deputy ministers, directors and policy analysts. Questions for these individuals also probed their general knowledge and perceptions of Lyme disease, and also probed a political party’s stance on Lyme disease and existing government policy. Questions regarding diagnosis and treatment options were only present in the family physicians and nurse practitioners’ survey. Lastly, the survey was translated in French by the co-author, Mario Levesque, a native Francophone, for administration. This is important given the sizable population of Francophones in New Brunswick at 31.6% (232,450 people) with smaller populations in Nova Scotia (3.3%, ~30,000 people) and PEI (3.7%, 4865 people) [50,51]. Once done, the survey was uploaded to Survey Monkey along with additional project information. No Lyme disease patients were surveyed for this study as the focus was on family physicians, nurse practitioners, provincial politicians and government health officials. 

### 2.3. Participant Identification

The identification of survey participants and, in particular, obtaining contact information (e-mail addresses) was challenging. This was especially the case for family physicians because while their telephone and facsimile numbers were listed on provincial health websites, e-mail addresses were not. To address this, a multipronged approach was used. First, a facsimile campaign was used for family physicians who were targeted based on their area of practise in relation to tick populations. Hence, for New Brunswick, the contact information for all family physicians was gathered for Health Regions 1 (Moncton/Southeast area), 2 (Saint John/South-West), and 6 (Bathurst/Northeast). The contact information for Region 4 (Edmundston/Northwest area) was also gathered as a potential control region, as tick populations are comparatively low in this region. Nova Scotia’s health system is not based on a regional system so certain geographic areas were selected to act in a similar fashion given known high tick populations. The first area included Halifax, Dartmouth, and Bedford; the second included Yarmouth, Shelburne, and Liverpool in the province’s southwest; and the third area included Pictou, Trenton, New Glasgow, and Antigonish (Northeast). Finally, for PEI, due to its smaller population (142,907 [51]) and geographic size, the contact information for all of the province’s family physicians was gathered. Our goal was to fax approximately half of the family physicians identified in the high tick population areas as shown in Table 1. 

Admittedly, a fax campaign is not the best approach given current technology with e-mail communication being the norm. We surmised that few family physicians would respond to a facsimile and were proven correct as only four (4) responses were received. Initially, we had tried to directly call the offices of family physicians to obtain their e-mail address, so we could forward them our information directly, but these efforts were futile as the vast majority of doctors’ offices claimed to either be uninterested or to rely primarily on fax as opposed to e-mail. We questioned this yet decided to proceed with the fax campaign, which had its own challenges. It was time consuming and more problematic was the fact that individuals were not able to simply click a hyperlink for the survey but would instead receive the fax and, to complete the survey, had to manually type in the survey URL from the fax. No doubt this had a negative effect on the response rate from family physicians.

To increase the response rate, provincial medical associations were contacted via e-mail and asked if they could forward the information letter and survey link to their members. This included all three nurse practitioners’ associations in the Maritimes, Doctors Nova Scotia and all three provincial Colleges of Family Physicians. All three nurse practitioners’ associations agreed to forward our materials to its members as did the New Brunswick and Nova Scotia Colleges of Family Physicians. Doctors Nova Scotia declined to do so while there was no response from the PEI College of Family Physicians. In addition, the survey was broadened to include the rest of Canada and was forwarded to various health professional associations for distribution to its members. For example, the Registered Nurses Association of Ontario endorsed our survey and distributed it to its members.

For provincial politicians and government health officials, obtaining their contact information was straightforward via government websites. In terms of government officials, individuals were largely selected from within the provinces’ Departments of Health based on their role including public health officers, deputy ministers, directors and policy analysts. This worked well for New Brunswick and PEI, but Nova Scotia’s website was less user friendly, which in turn resulted in less contact information for Nova Scotian government officials and therefore less officials selected for the province as shown in Table 2.

### 2.4. Response Rates

In total, the provincial politicians and government health officials survey was sent to 152 individuals across the Maritimes with nine (9) completed surveys for a response rate of 5.92% which is considered low. Of the nine completed surveys, 4 were from NB, 4 from NS, and 1 from PEI. The family physicians and nurse practitioners survey was distributed to 1833 family physicians (949 in New Brunswick, 884 in Nova Scotia) via facsimile or through their associations. It is unknown if it was distributed to family physicians in PEI (102) by its College of Family Physicians as noted above but it was faxed to half of them. In addition, it was distributed to 286 nurse practitioners via their provincial associations (127 in New Brunswick, 124 in Nova Scotia and 35 in PEI). All told the survey was distributed to 2171 family physicians and nurse practitioners with 40 surveys completed (4 in response to the fax campaign), and all 40 completed surveys were from family physicians (i.e., no responses were received from nurse practitioners). This represents a response rate of 1.84% which is very low. 

This brings up a host of questions related to the response rate. First, a fax campaign is not a good method for survey administration given the state of technology as previously discussed. Second, the medical professions are a closed network making it very difficult to access and gain participation, especially for social science researchers. Still, two of the three Colleges of Family Physicians (NB, NS) did agree to distribute our survey as did the nurse practitioners’ associations, which helped especially since the fax campaign led to poor results. Even so, soliciting participation via association newsletters is not the same as directly contacting someone. A third issue was the time of year the survey was administered—late June and July 2018, summer months—with July especially being a time of the year when many people typically take holidays. This was hard to avoid since the project began 1 May 2018 and had to be completed by 31 August 2018. Time was required to draft the ethics proposal and to obtain research ethics approval (granted at the end of May 2018). No doubt the time of year contributed to the lower response rate. Lastly, the Office of the Chief Medical Officer of Health for New Brunswick in conjunction with the New Brunswick Medical Society completed a survey on Lyme disease exploring physicians’ clinical experience and management of the disease in 2016 [42]. While the results of this survey were not released until late July 2018, it is possible that our survey, while different in orientation, was seen as duplication and therefore dismissed by New Brunswick family physicians thus negatively affecting our response rate. For example, our response rate from New Brunswick family physicians was 3.0% (28/949) which is one-fifth the response rate of the NB Medical Society 2016 survey of 16.1%. This, however, does not explain the low response rate from Nova Scotia at 1.2% (11/884) and the 0% response rate from PEI family physicians. The PEI case is also interesting given the series of questions received from government officials as to the publication of our results and the type of responses desired. We wonder if this had a chilling effect on survey completion for PEI. Overall, of the 40 completed surveys, 29 were from New Brunswick, 11 from Nova Scotia. There were no responses from PEI. Given the low response rates, the results should be treated as strictly exploratory.

## 3. Results and Discussion

### 3.1. Key Points from the Surveys

Three key points emerged for the surveys concerning the prevalence and knowledge of Lyme disease and government action. 

#### 3.1.1. Prevalence of Lyme Disease

**Figure A1:**
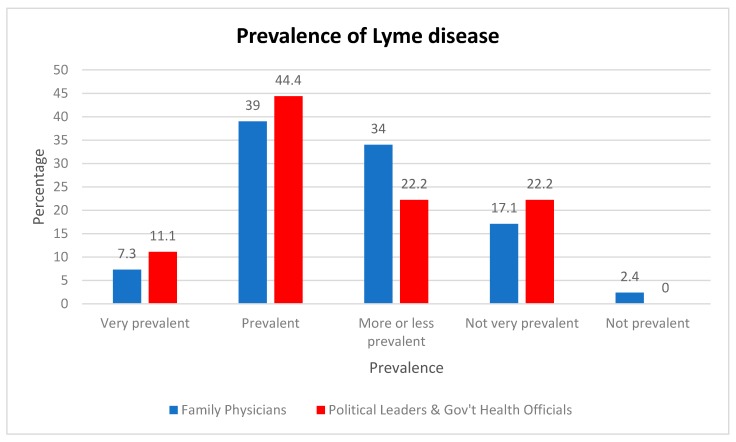
Responses from family physicians, political leaders and government health officials to the survey question: In your opinion how prevalent is Lyme disease in the Maritimes?

First, in terms of prevalence and shown in Figure A1, the vast majority of respondents identified that Lyme disease is at least somewhat prevalent, with 46.3% of family physicians stating the disease was either ‘very prevalent’ or ‘prevalent’ and 34% stating the disease was ‘more or less prevalent’. Similar responses were noted among political leaders and government health officials with 55.5% answering ‘very prevalent’ or ‘prevalent’. Combined, the results demonstrate that most family physicians and political leaders and government health officials are aware that Lyme disease affects their region within Canada. 

**Figure A2:**
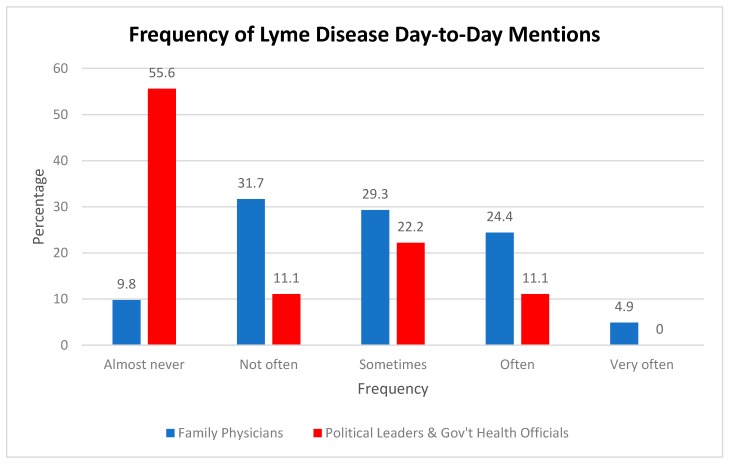
Responses from family physicians, political leaders and government health officials to the survey question exploring how often Lyme disease is mentioned in their day-to-day activities.

**Figure A3:**
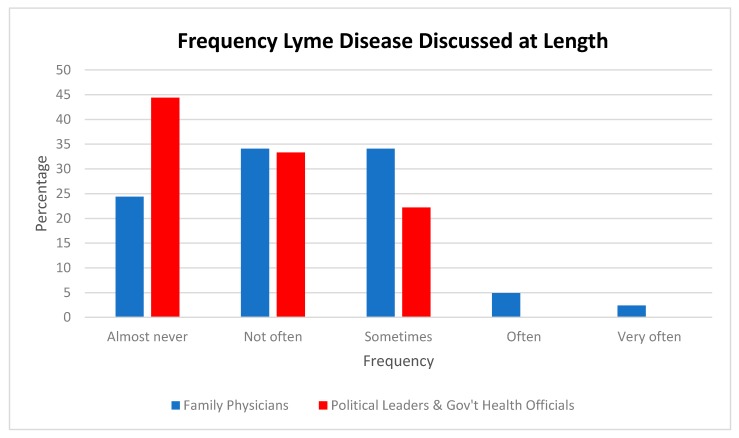
Responses from family physicians, political leaders and government health officials to the frequency with which Lyme disease is discussed at length in their day-to-day work.

**Figure A4:**
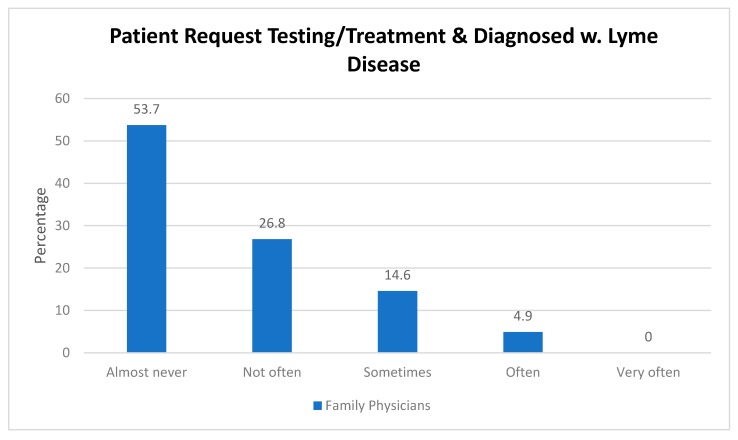
Responses from family physicians identifying the percentage of patients that have requested testing or treatment been diagnosed with Lyme disease.

However, knowledge of Lyme disease’s prevalence did not translate into a significant topic of discussion for respondents. As shown in Figure A2, 66.7% of political leaders and government health officials stated that Lyme disease was discussed ‘not often’ or ‘almost never’ in their day to day work. There was less agreement with family physicians, with 41.5% stating that Lyme disease was discussed ‘almost never’ or ‘not often’, 29.3% stating ‘sometimes’, and 29.3% stating that it was discussed ‘often’ or ‘very often’. The variance in responses may be accounted for by differences in tick populations and by extension Lyme disease prevalence across the Maritimes [8,44,45]. When asked how often the disease was discussed “at length”, that is, more than a brief word in passing, (Figure A3), 58.5% of family physicians chose ‘not often’ or ‘almost never’. This would imply that while some regions may experience more cases of Lyme disease than others, the majority of family physicians do not have Lyme disease at the top of mind. Furthermore, in terms of diagnosis (Figure A4), 80.5% of family physicians claimed that individuals showing symptoms that could be attributed to Lyme disease are ‘not often’ or ‘almost never’ diagnosed with Lyme disease. This could be interpreted multiple ways as most of the family physicians could argue this is due to Lyme disease having symptoms that overlap with many other conditions, while Lyme disease patients and patient advocates may argue that this reflects misdiagnosis. 

#### 3.1.2. Knowledge of Lyme Disease

**Figure A5:**
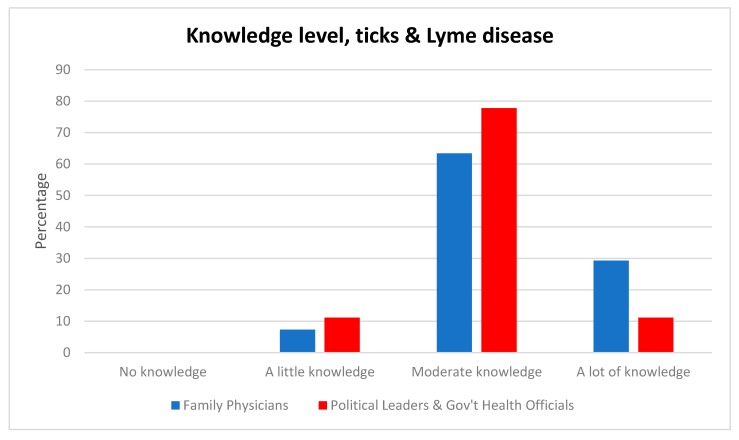
Responses from family physicians, political leaders and government health officials assessing their knowledge level of ticks and Lyme disease.

**Figure A6:**
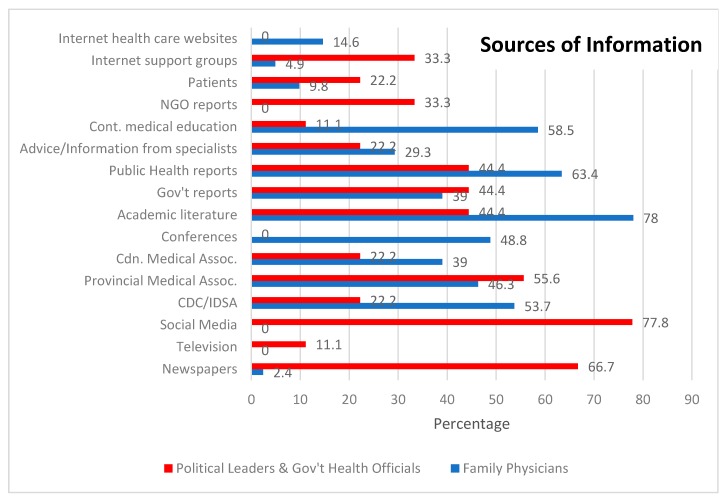
Responses from family physicians, political leaders and government health officials identifying their sources of information about ticks and Lyme disease.

**Figure A7:**
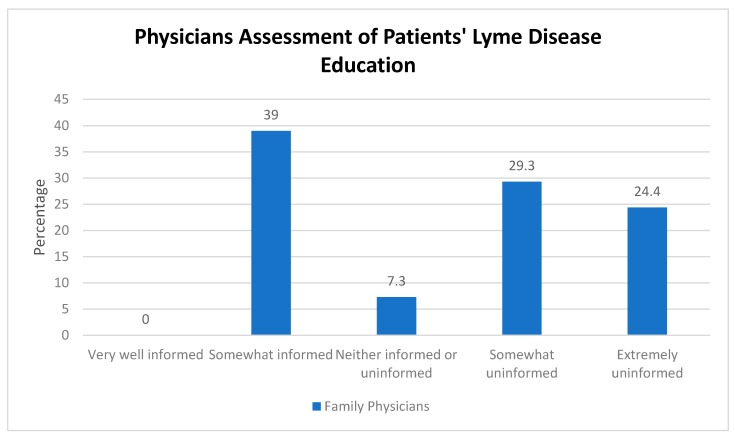
Responses from family physicians assessing how educated they felt their patients were about Lyme disease.

Second, in terms of knowledge about Lyme disease, 92.7% of family physicians and 88.9% of political leaders and government health officials claim to have ‘moderate’ to ‘a lot of knowledge’ on Lyme disease (Figure A5). When asked where they obtained their knowledge (Figure A6), the top three answers for family physicians were academic literature (78%), public health agency reports (63.4%) and continuing medical education (58.5%). Family physicians also questioned how well informed their patients were about Lyme disease with 39% responding that their patients were ‘somewhat informed’ while 53.7% stated that patients were ‘somewhat uninformed’ or ‘extremely uninformed’ (Figure A7). These results suggest a divide between a majority of family physicians and patients which is consistent with the literature. Political leaders and government health officials seem to be more sympathetic to the position of patients and patient advocates, commenting that they needed to “admit that there is a problem” and noting the need to “start testing and treating properly.” Such comments may be a result of where political leaders and government health officials obtained their information concerning Lyme disease, with 77.8% citing social media as their number one source of information followed by newspapers (66.7%) and provincial medical associations (55.6%) (Figure A6). The reliance on social media and newspapers as significant sources of information may be cause for concern depending on the quality of information posted. 

#### 3.1.3. Government Actions

**Figure A8:**
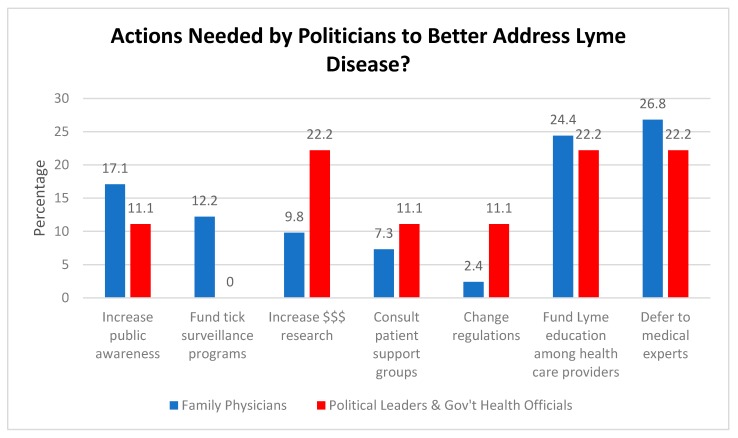
Responses from family physicians, political leaders and government health officials for what politicians could do to improve how Lyme disease is addressed.

Lastly, questions arise on the effectiveness of government actions to date, which is important in the context of this research. When asked to rank options provided in response to the question, “Which of the following could politicians do to improve how Lyme disease is addressed?”, the top three responses by family physicians were to ‘defer to medical experts’, ‘fund Lyme disease education among health care providers’ and ‘increase public awareness’ as shown in Figure A8. The responses from political leaders and government health officials largely mirror those of family physicians except that decision makers were much more interested in increasing research and not interested at all in funding tick surveillance programs. Combined, these results are reflected in the federal, New Brunswick, and Nova Scotian Lyme action plans. However, the lack of interest in tick surveillance identified by decision makers is problematic given the fact that surveillance programs are helpful in predicting increases in Lyme disease cases.

While identified policy options have been established as priorities by governments, most family physicians do not feel these efforts have influenced their approach to Lyme disease. For example, 82.9% of medical professionals and 100% of political leaders and government health officials felt that the *Federal Framework on Lyme Disease* had resulted in either ‘no changes’ or had not played a role in any change to approaching Lyme disease. Furthermore, 58.6% of family physicians feel that increased federal funding to Lyme research had not affected their approach to Lyme disease, while 77.8% of provincial political leaders and government health officials had also felt no affect. This ultimately brings into question the value of current federal and provincial government approaches to Lyme disease as some of their key priorities included educating and implementing best practise guidelines among physicians who many had felt were not properly educated in terms of Lyme disease. However, the majority of responding family physicians in New Brunswick, Nova Scotia and PEI have not been affected by these government efforts. 

### 3.2. Challenges Identified

**Figure A9:**
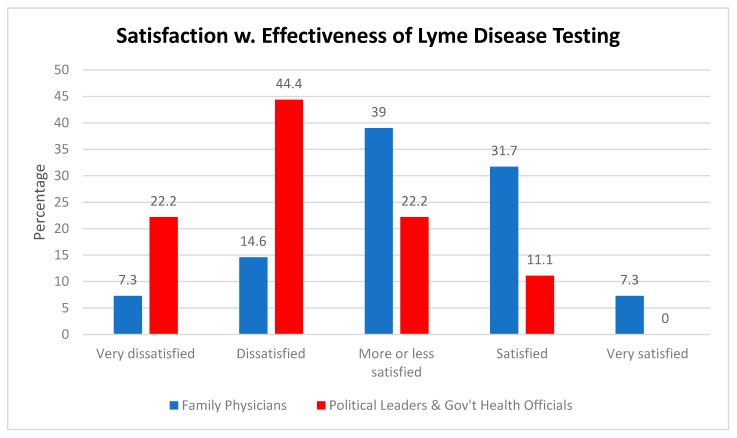
Responses from family physicians, political leaders and government health officials assessing their satisfaction levels with the current available testing for Lyme disease.

The survey findings demonstrate debates prevalent in the literature are also prevalent within the Maritimes: Many are unhappy with the current medical practises, the media is critical of the health care system, and a significant portion of family physicians feel their patients are misinformed [34]. Furthermore, 61% of family physicians identified that they were aware of patients seeking treatment in the United States, yet family physicians remained satisfied with current testing protocols (Figure A9). This demonstrates that the pushes and pulls away from the mainstream Canadian health care system identified by Boudreau, Lloyd, and Gould are also significant specifically within the Maritimes [10]. 

The survey results also identified the minimal effectiveness of government policies and action plans. For example, political leaders and government health officials noticed no change in how the disease is approached, meaning there could not have been noticeable changes in the realm of education, surveillance or public awareness. This is important because it is patients and patient advocates that argue that family physicians are poorly educated about Lyme disease. The fact survey results highlight family physicians have not changed their approach to Lyme disease given government actions is concerning. We question whether this is ineffective government action or whether there is a time lag effect at play. That is, government Lyme disease frameworks, policies and action plans are relatively new, most within the last decade, and more time is needed for their effects to be realized. 

One of the biggest blockages remains current best practise guidelines for the diagnosis and treatment of Lyme disease. The much-debated guidelines dictate the diagnosis process stating that an individual can only be diagnosed clinically if the patient shows a bull’s eye rash and had plausible exposure to an area with known tick populations. If they are showing specific symptoms and do not have a rash then the two-tiered serological testing is undertaken [9]. This protocol has been criticized by Ogden et al. who voiced concerns over clinical diagnosis being weighted towards plausible exposure rather than the patients’ clinical condition, while Boudreau, Lloyd, and Gould have voiced their concerns that geographic variation in Lyme disease may be resulting in false negatives in the serological testing [1,10]. Yet, family physicians within the Maritime Provinces are content with the current testing process as our survey results reveal. 

Similarly, the debate surrounding chronic Lyme disease and the length of antibiotic treatment is linked to the guidelines, as antibiotics are to be kept under a month [17]. These guidelines are the central blockage facing individuals who feel they are struggling to receive treatment within the Maritimes as they dictate the testing and treatment options one receives. Family physicians are well aware of this blockage as the survey revealed their knowledge of many patients seeking treatment in the U.S. For patients and patient advocates, a review of current clinical guidelines is required, preferably conducted by an independent third party that includes substantive and meaningful patient engagement [52,53]. While the medical community has increasingly yet slowly included patients in research and meetings, care is required to ensure that such engagement moves beyond tokenism [54,55,56,57].

### 3.3. The Politics of Lyme Disease 

Understanding the relationships between problems, policies and politics (decision-making) remains crucial and is at the heart of John Kingdon’s multiple streams framework of policy making, which is used below to assess the politics of Lyme disease [58,59]. Kingdon’s framework introduces randomness and ambiguity to the decision-making process to examine which issues get addressed by decision makers. Given time constraints, few issues get their attention, and for those that do, accepted solutions are less than optimal. This should come as no surprise, as Kingdon notes that solutions and problems act independently. Multiple solutions and problems exist based on imperfect information held among a diverse set of stakeholders who find it increasingly difficult to agree on a path forward. The contested science surrounding the diagnosis and treatment of Lyme disease among and between the medical community, government decision makers, patients and patient advocates illustrates this nicely. Ambiguity remains prominent in the decision-making process.

Three processes are at the heart of the multiple streams framework. Problems are issues in need of attention. They come into focus based on indicators that measure conditions, due to focusing events, or through the study of issues. The increased spread of ticks and cases of Lyme disease along with more people seeking treatment outside of the Canadian health care system are problems in need of attention. Policies are ideas for how to address identified problems. Ideas are plentiful and vary depending on the stakeholder, with few seriously considered by decision makers because of time and institutional constraints, among other things. Lastly, politics refers not only to the individuals involved in the policy process (and their turnover), but also to the broader set of groups (e.g., patient advocates) exerting pressure for change as well as to the political climate within which they operate (i.e., ideology).

The key to policy making in the multiple streams process is the coupling of problems, policies and politics. All three need to come together for an issue to be addressed, and this is where much of the randomness occurs. For example, problems and policies coming together in an inhospitable political climate will go nowhere. For the three streams to come together, a window of opportunity must open for coupling to occur. These can be random opportunities, such as a favourable minister or deputy minister entering the decision-making process or a change in attitudes of family physicians for the diagnosis and treatment of patients. Alternatively, windows of opportunity can be regular, such as through yearly budget consultation processes or regular elections. The coupling of the streams also needs to be facilitated by policy entrepreneurs. These are highly skilled individuals attuned to the political climate and cognizant of existing problems and policies. With their substantial resources, they can bring the streams together by crafting an acceptable framework for stakeholders to support.

The multiple streams framework is useful for assessing decision making by considering the randomness and ambiguity involved in the process [59]. It is used here to shed light on the current Lyme disease situation in Canada’s Maritime provinces. The most recent provincial and federal plans on tackling Lyme disease came after a period of significant rise in Lyme disease. In 2009, there were 130 reported cases of Lyme disease in Canada but by 2015 there were more than 700 [60]. Furthermore, there was ample scholarship linking climate change and tick expansion, which projected Lyme disease expansion in Canada [5]. These factors helped shape a narrative that Lyme disease was expanding in Canada. As more stories and public concern surrounding Lyme disease and its treatment grew through media stories and advocacy groups, politics gradually shifted to benefit an active approach to Lyme disease. Finally, when consulting medical professionals and experts, the consensus seemed to be education, surveillance and research, which has been reflected in the federal, New Brunswick, and Nova Scotian action plans, as well as subsequent Lyme disease policy. However, this begs the question: Why are tensions still so high when it comes to Lyme disease and why is this policy approach falling short? The fact governments undermine their own efforts, as the comments of Dr. Robert Strang, Nova Scotia’s Chief of Public Health demonstrates, has not helped matters.

One of the main issues is defining the problem itself. There has been little convergence between the medical community, patients and patient advocates on how to address outstanding issues. The medical community generally does not see Lyme disease as anything other than an emerging disease. Defining the problem in this manner places an emphasis on tracking tick populations and keeping track of endemic regions. However, much of the discontent that has been cited in the media, as well as some scholarship [10], surrounds issues with the diagnostic process and available treatment. Since there is no agreement on the problem, in particular the science at the heart of the issue, it is hard for policy to address an ambiguous problem. 

Differing perspectives on the problem of Lyme disease is also evident in the current policy on Lyme disease. These policies seem to be addressing the fact that tick populations, and by extension Lyme disease, are on the rise and are predicted to continue. This reflects government policy aligning with public health researchers, who emphasize endemic regions and the expansion of tick populations. This is reflected in the focus on tick surveillance and minimal policy addressing the main concerns of diagnosis and treatment. Furthermore, most experts who inform policy stand by the current protocol as being the safest option for Lyme disease, making it even less likely that policy would touch on the concerns of patients and patient advocates. Even as the politics stream may be slightly in favour of patients and patient advocates at the moment, the problem remains unsettled thereby undercutting needed policy actions from emerging. In short, the three streams are out of alignment.

There are some factors that could affect this gridlock as multiple streams theory claims that events may bring a problem to the forefront of the policy agenda or change how the issue is perceived. In terms of a potential crisis, Eastern Canadian tick populations may expand to 80% of human inhabited areas by 2020 [5]. Such circumstances may strain the conventional health care system and force governments to perceive the problem of Lyme disease in an urgent and critical manner. Conversely, discoveries in either testing or treatment may be able to bridge the gap between the medical community, patients and patient advocates. This has some merit as new Lyme disease vaccines are currently entering the testing phase and may be publicly available in the next five years [61]. Regardless, there is still a great deal that can be currently done to effect change, particularly through the role of policy entrepreneurs.

For a policy entrepreneur looking to push policy that addresses the current clinical guidelines concerning Lyme disease, one of the first actions would be to change the perception of this issue within the medical community. Admittedly, this may have to happen internally given the resistance of the medical community to outsiders, yet we note the gradual increase of patient voices in research and meetings as a positive trend to that end. Policy entrepreneurs can also seek to address the source of the Canadian guidelines given they were not created domestically but by the Infectious Diseases Society of America (IDSA) and Centers for Disease Control (CDC) [42]. We note considerable conflicts of interest when it comes to these organizations that may undercut the legitimacy of their recommendations. The IDSA, for example, is a private medical society that has created guidelines for Lyme disease, while at the same time many CDC officials responsible for Lyme disease policy are also members of the IDSA [62]. Thus, it is little coincidence that the CDC has been exclusively endorsing the IDSA recommendations. To make matters worse, U.S. government officials can personally benefit from patents they create with taxpayer dollars. Therefore, a CDC official can benefit from their Lyme disease related patent while operating in a position that is responsible for assessing new technologies that may be in competition with their own patent [62]. There is a considerable amount of supporting evidence that makes this potential conflict of interest even more incriminating [62,63]. Furthermore, many of the authors of the 2006 IDSA guidelines hold prominent professional roles with significant government funding and influence within the dominant medical journals, often blocking articles that may present contrary conclusions to their own [62]. These facts—potential conflicts of interest, lack of transparency, concerns over guidelines development and key roles of many individuals noted above—question the validity of the CDC and IDSA and their guidelines. Focusing on these organizations more specifically may make it possible to change the Canadian medical community’s perception on their current guidelines. 

While a critical focus on the CDC may help change the perception of Lyme disease among experts, there would still need to be general agreement in terms of a policy option. These options often must adhere to the general norm and tendencies of the government to help in it being perceived as reasonable [59]. Considering this, a feasible policy option may be to strike an independent federal Commission to review the current Lyme disease guidelines. This option would not necessarily discredit the current stance of the medical community and may make the policy an easier sell to medical experts. A priority of this Commission would be to bridge the gap between patients of Lyme disease and experts and thus would bring government officials, medical professionals, patients and patient advocates to the table throughout the process. Open, unbiased dialogue that includes and validates the experiences of all stakeholders is required. Forming a Commission to facilitate this dialogue may help in bringing the streams together for substantive policy action to emerge.

## 4. Conclusions

This project examined the current state of Lyme disease within Canada’s Maritime provinces (New Brunswick, Nova Scotia, Prince Edward Island) from a political perspective, a subfield that is still relatively new to the study of Lyme disease. It identified the ongoing debates concerning Lyme disease in the literature and, through surveys, revealed their prevalence in the Maritime provinces. Given the study’s low response rate, the findings should be considered exploratory and not conclusive. They are nonetheless valuable given it is one of the first attempts to think critically about Lyme disease policy in the region. 

Ultimately, Lyme disease is a Pandora’s Box of debates and ambiguity. Both the medical community and patient advocates do not seem poised to make any concessions soon. However, a considerable amount of scholarship is projecting the dramatic spread of tick populations to continue, suggesting that cases of Lyme disease will only continue to grow. Whether you agree with Lyme disease patients and patient advocates or not, when over half of the family physicians responding in our survey were aware that they have patients seeking treatment in the U.S., something must be wrong with the current diagnosis and treatment options in Canada. As advancements in diagnostic and or treatment technology still seem to be years away, action is needed to address the concerns of patients, patient advocates and former Lyme patients as their numbers are only projected to grow. Our recommendation of an independent Commission of inquiry that brings together government officials, medical professionals, patients and patient advocates is a reasonable way of fostering understanding and exploring policy options. 

## Figures and Tables

**Table 1:** Number of Family Physicians Identified for this Study, by Province and Region.

Province	No. Identified	No. with Fax #	No. Faxed	No. Failed After 2 Tries	Net No.
New Brunswick					
Health Region 1 (Moncton)	283	196	100	10	90
Health Region 2 (Saint John, Southwest NB)	199	110	80	3	77
Health Region 6 (Bathurst)	91	58	58	0	58
Health Region 4 (Edmundston)	69	44	44	0	44
NB Totals	642	408	282	13	269
Nova Scotia					
Halifax, Dartmouth, Bedford	313	226	92	3	89
Yarmouth, Shelburne, Liverpool	28	19	19	0	19
Pictou, Trenton, New Glasgow, Antigonish	36	17	17	0	17
NS Totals	377	262	128	3	125
Prince Edward Island	102	102	52	0	52
PEI Totals	102	102	52	0	52
Total	1121	772	462	16	446

**Table 2:** Number of Provincial Politicians, Government Health Officials, by Province Selected for Survey Completion.

	New Brunswick	Nova Scotia	Prince Edward Island (PEI)	Total
Provincial Politicians (MLAs)	49	51	27	127
Government Health Officials	14	6	5	25
Total	63	57	32	152

## Supplementary Materials

The following are available online at https://www.mdpi.com/1660-4601/16/9/1531/s1, File 1: Lyme Disease in the Maritimes Survey.
