# Supplementary material for: A Multiple Streams Approach to Understanding the Issues and Challenges of Lyme Disease Management in Canada’s Maritime Provinces
Source: Int J Environ Res Public Health. 2019 Apr 30;16(9):1531. doi: 10.3390/ijerph16091531 (PMC6539885; doi:10.3390/ijerph16091531)
Supplement: Supplementary file 1 [file ijerph-16-01531-s001.pdf]

LYME DISEASE IN THE MARITIMES SURVEY

The purpose of this research project is to identify potential challenges present within the health care system for the treatment of Lyme disease, specifically within the Maritimes, and to craft policy options to address them.

Your participation in this research project is voluntary. You may choose not to participate. If you decide to participate in this study you may withdraw at any time with no penalty.

The procedure involves filling out this online survey which should take approximately 15 minutes to complete. Any comments provided may be quoted anonymously in this project's final report. Your responses will be kept confidential and data will be password protected.

You will be asked questions about the prevalence of Lyme disease, the process of diagnoses, diseases that can be mistaken for Lyme disease, training for diagnosing Lyme disease, and services available to those who have contracted Lyme disease.

This research has been reviewed and approved by the Mount Allison University Research Ethics Board. If you have any questions or concerns about this study, you may contact the Chair, ___________________, by phone (XXX-XXX-XXXX) or by e-mail at ________@______.

Please note that the online survey is hosted by "Survey Monkey" which is a web survey company located in the USA. All responses to the survey will be stored and accessed in the USA. This company is subject to U.S. laws, in particular, to the U.S. Patriot Act that allows authorities access to the records of Internet service providers. If you choose to participate in the survey you understand that your responses to the questions will be stored and accessed in the USA. The security and privacy policy for Survey Monkey can be viewed at https://www.surveymonkey.com/

COMPLETION OF THIS SURVEY WILL BE TAKEN AS CONSENT.

Questions? Contact Matthew at mdklohn@mta.ca or Mario at malevesque@mta.ca.

1. Please select your current profession.

- Physician / Medical Professional
- Government Official
- Policy Analyst
- Health Authority Official
- Politician
- NGO

2. In which of the Maritime Provinces do you practise/are located?

- Prince Edward Island
- New Brunswick
- Nova Scotia

3. In your opinion how prevalent is Lyme disease in the Maritimes?

- Very prevalent
- Prevalent
- More or less prevalent
- Not very prevalent
- Not prevalent at all

4. In your day to day work how often is Lyme disease *mentioned*?

- Almost never
- Not often
- Sometimes
- Often
- Very often

5. In your day to day work how often is Lyme disease *talked about at length*?

- Almost never
- Not often
- Sometimes
- Often
- Very Often

6. How often have patients that have requested testing or treatment actually been diagnosed with Lyme disease?

- Almost never
- Not often
- Sometimes
- Often
- Very often

7. What is your level of knowledge about ticks and Lyme disease?

- No knowledge
- A little knowledge
- Moderate knowledge
- A lot of knowledge

8. Where do you obtain your knowledge about ticks and Lyme disease?
Check all that apply:

- Newspapers
- Television
- Social Media (Twitter, Facebook etc.)
- CDC/IDSA
- Provincial medical associations
- Canadian Medical Association
- Conferences
- Academic literature
- Government reports
- Reports from Public Health
- Advice/information from specialists
- Continuing Medical Education
- NGO reports
- Patients
- Internet support groups
- Internet health care websites

Other health care information (please specify)

9. How well informed are your patients about Lyme disease?

- ◯ Very well informed
- ◯ Somewhat informed
- ◯ Neither informed or uninformed
- ◯ Somewhat uninformed
- ◯ Extremely uninformed

10. From your experience, which of the following could *politicians* do to improve how Lyme disease is addressed? Please rank the options:

Increase public awareness	▾
Fund tick surveillance programs	▾
Increase funding for tick/Lyme disease research	▾
Consult patient support groups	▾
Change regulations	▾
Fund Lyme education for health care providers	▾
Defer to medical experts, this is a medical issue	▾

11. In 2015, Bill C-442 established a 'Federal Framework for Lyme disease'. Has this framework and or subsequent conferences and government reports influenced your approach to Lyme disease?

- ◯ Greatly influences
- ◯ Somewhat influences
- ◯ No changes
- ◯ Approaches have changed but not as a result of these efforts

12. In 2017 the Federal government set aside $4 Million to Lyme disease research, in your experience has this affected how Lyme disease is being approached?

- ◯ Greatly influenced
- ◯ Somewhat influenced
- ◯ No Change
- ◯ Approaches have changed but not as a result of these efforts

13. Are you aware of any of your patients seeking treatment for Lyme disease in the United States?

- Yes
- No

14. Overall, how satisfied are you with the effectiveness of available testing for Lyme disease?

- Very dissatisfied
- Dissatisfied
- More or less satisfied
- Satisfied
- Very satisfied

15. Any other comments concerning Lyme disease and or the survey:

Thank You!
